# Health Personnel’s Perceived Usefulness of Internet-Based Interventions for Parents of Children Younger Than 5 Years: Cross-Sectional Web-Based Survey Study

**DOI:** 10.2196/15149

**Published:** 2020-11-18

**Authors:** Hege Therese Størksen, Silje Marie Haga, Kari Slinning, Filip Drozd

**Affiliations:** 1 Department for Infant Mental Health Regional Centre for Child and Adolescent Mental Health, Eastern and Southern Norway Oslo Norway

**Keywords:** internet, parent support, children, mental health, acceptability, health care services

## Abstract

**Background:**

Approximately 10%-15% of children struggle with different socioemotional and psychological difficulties in infancy and early childhood. Thus, health service providers should have access to mental health interventions that can reach more parents than traditional face-to-face interventions. However, despite increasing evidence on the efficacy of internet-based mental health interventions, the pace in transferring such interventions to health care has been slow. One of the major suggested barriers to this may be the health personnel’s attitudes to perceived usefulness of internet-based interventions.

**Objective:**

The purpose of this study was to examine health professionals’ perceived usefulness of internet-based mental health interventions and to identify the key areas that they consider new internet-based services to be useful.

**Methods:**

Between May and September 2018, 2884 leaders and practitioners of infant and child health services were recruited to a cross-sectional web-based survey through the following channels: (1) existing email addresses from the Regional Centre for Child and Adolescent Mental Health, Eastern and Southern Norway, course database, (2) an official mailing list to infant and child health services, (3) social media, or (4) other recruitment channels. Respondents filled in background information and were asked to rate the usefulness of internet-based interventions for 12 different infant and child mental health problem areas based on the broad categories from the Diagnostic Classification of Mental Health and Developmental Disorders of Infancy and Early Childhood (DC:0-5). Perceived usefulness was assessed with 1 global item: “*How often do you think internet-based self-help programs can be useful for following infant and child mental health problems in your line of work?*” The answers were scored on a 4-point scale ranging from 0 (*never)* to 3 (*often*).

**Results:**

The participants reported that they sometimes or often perceived internet-based interventions as useful for different infant and child mental health problems (scale of 0-3, all means>1.61). Usefulness of internet-based interventions was rated acceptable for sleep problems (mean 2.22), anxiety (mean 2.09), and social withdrawal and shyness (mean 2.07), whereas internet-based interventions were rated as less useful for psychiatric problems such as obsessive behaviors (mean 1.89), developmental disorders (mean 1.91), or trauma (mean 1.61). Further, there were a few but small differences in perceived usefulness between service leaders and practitioners (all effect sizes<0.32, all *P*<.02) and small-to-moderate differences among daycare centers, well-baby clinics, municipal child welfare services, and child and adolescent mental health clinics (all effect sizes<0.69, all *P*<.006).

**Conclusions:**

Internet-based interventions for different infant and child mental health problems within services such as daycare centers, well-baby clinics, municipal child welfare services, and child and adolescent mental health clinics are sometimes or often perceived as useful. These encouraging findings can support the continued exploration of internet-based mental health interventions as a way to improve parental support.

## Introduction

### Background

Children aged 0-5 years depend on their relationship with their primary caregiver for survival and development. The main task of the parents during these years is to be socially and emotionally available, identify and understand their child’s needs, and respond to his or her needs appropriately [1]. For most parents, these parenting skills are intuitive. However, during the many and frequent developmental shifts in the child’s early years of life, most parents will find some to be more challenging than others. Children may struggle with different socioemotional and psychological difficulties such as sleep problems [2], excessive crying, or aggressive behavior, which present opportunities for unhealthy developmental pathways in children, which in turn can affect the parent-child relationship, including increased risk of neglect and violence. These are problems that the child may carry with him or her into adolescence and young adulthood. Therefore, primary prevention and early intervention in terms of parental support or training programs, wherein the purpose is to strengthen parenting and parent-child relationship and ensure children’s right to care and protection may be highly beneficial for preventing early life difficulties turning into longer lasting problems such as mental illness and their potential consequences [3].

Epidemiological research with children younger than 3 years is limited, but a few studies have indicated prevalence rates of 10%-15% of subclinical/clinical symptoms of mental health disorders [4,5]. These prevalence rates seem to increase from the age of 3 years, as it is estimated that 15%-20% of older children have a reduced functioning due to symptoms most commonly associated with anxiety, affective, and behavioral disorders [6]. Approximately 7%-8% of preschool and school-aged children have symptoms that are compatible with a psychiatric diagnosis at the time of examination [7,8].

Systematic reviews have shown that face-to-face parenting interventions can be effective for children with severe attachment problems [9] and internalizing [10] and externalizing problems [11]. However, to effectively reach many parents and potentially target a broader range of problems, it is necessary to exploit the inherent characteristics of modern technology such as the internet that has a high reach at low cost. Internet-based interventions can also help reduce geographical and social inequalities in health care by providing web-based access for patients to health treatments in remote and rural areas that may lack treatment options and trained health care providers. Internet-based interventions can reach many parents, are in line with their preferences for self-help, have few side effects and high response rates, and can increase the capacity of health care professionals and cover some of the lack of trained personnel [12].

There have been relatively few studies on internet-based parenting programs among families with children aged 0-3 years. Meta-analytic studies suggest that digital cognitive-behavioral programs can be effective for children from the age of 3 years and upwards for targeting depression and childhood anxiety [13,14], disruptive behaviors [15], and somatic conditions [16]. It is therefore reasonable to think that digital interventions may be useful for children younger than 3 years as well. However, there are several barriers to the adoption of new treatments by professionals into their daily practice and few available and accessible e-mental health solutions [17]. Increased availability of e-mental health solutions itself can be positive for health personnel’s knowledge and acceptability of digital solutions [18]. However, this is unlikely to be sufficient for uptake and sustained use. In addition to the availability of new digital services, one of the major barriers to their uptake is clinicians’ attitudes [17,19].

Attitudes and acceptability toward information technology, conceptualized as perceived usefulness, has, over the years, been one of the major determinants of usage intentions [20,21]. Researchers have proposed and tested several models to explain and predict user acceptance and use of information technology. In 2003, Venkatesh et al [21] integrated elements from these acceptance models to create the Unified Theory of Acceptance and Use of Technology (UTAUT), which identifies 4 key factors (ie, performance expectancy, effort expectancy, social influence, and facilitating conditions) and 4 moderators (ie, age, gender, experience, and voluntariness) related to describe the processes underlying the development and change of attitudes toward digitally delivered interventions primarily in organizational contexts. There are several similar constructs pertaining to user acceptance identified in other models; hence, the term “performance expectancy” is used interchangeably with “perceived usefulness.” These similar concepts confirm from different angles that perceived usefulness plays an important part in forming users’ attitudes or behavioral intentions.

Despite limited research on child health practitioners (and parental) user acceptance and opinions of e-mental health services, there are still a few studies available; one of these studies showed that obstetricians may be skeptical toward the use of eHealth solutions [22]. However, child and youth mental health workers seem cautiously positive [23,24], particularly in the prevention and treatment of mild-to-moderate mental health problems [24,25]. Furthermore, previous research has shown that leadership and different leadership styles may also have an impact on the adoption and perceived usefulness of technology [26,27]. Thus, it is necessary to consider perceived usefulness from the perspective of different professions/health care services for young children and professional roles (eg, practitioners vs leaders), which, to the best of our knowledge, has not been previously studied directly. In summary, it is therefore important to examine health professionals’ perceived usefulness of e-mental health programs and identify the key areas in which they consider new e-mental health services to be useful.

### Aims of This Study

The aim of this study was to examine how often health personnel in prenatal, infant, and child health care services for children younger than 5 years would find internet-based parent support interventions useful for different target groups and problem areas. More specifically, we wanted to examine if there are differences in the perception of the usefulness of e-mental health intervention between leaders, middle managers, and practitioners and among daycare centers, well-baby clinics, municipal child welfare services (CWS), and child and adolescent mental health clinics (CAMHS).

## Methods

### Study Design and Participants

This study was conducted as a cross-sectional web-based survey and approved by the Norwegian Centre for Research Data [28]. We aimed to assess the views of all types of infant and child health practitioners. Participants who were 16 years or older were recruited either through any of the following channels: (1) existing email addresses from the Regional Centre for Child and Adolescent Mental Health (RBUP), Eastern and Southern Norway, course database, (2) an official mailing list to infant and child health services, (3) social media or (4) other recruitment channels (ie, national labor unions). Administrative staff was excluded from this study.

We extracted 5050 unique email addresses from the course database at the time of recruitment based on participation in supplementary education and courses for leaders and clinical staff working with parents and children younger than 5 years. Owing to ethical considerations, participants who registered in the course database before January 2013 and with a private email address were not invited to participate in the study (1448/5050, 28.7%). This was in line with Norwegian ethical codes. Thus, 3602 (71.3%) unique and eligible participants received a study invitation.

The official mailing list [29] contained 7345 unique official email addresses to relevant infant and child mental health services. These included the leaders and clinical staff in the following primary care services: (1) municipal services (n=590), (2) state CWS (n=22), (3) daycare centers (n=5627), (4) family counselling services (n=59), (5) district medical officers (n=190), (6) leading public health nurses (n=247), (7) municipality psychologists (n=108), and (8) educational and psychological counselling services (n=89). The following secondary care services were also included: leaders and clinical staff in CAMHS (n=178) and maternity wards in hospitals (n=20). Finally, 215 email addresses were to other services (eg, private practices and low-threshold services).

Participants were also recruited by promoting the study in 14 relevant Facebook groups with a total of 16,555 group members. These were, however, not unique members (ie, a participant may be a member of more than one group). In addition, 3 national labor unions, that is, the Norwegian Midwife Association, Norwegian Association for Clinical Pedagogy, and the Norwegian Psychologist Association were contacted by mail or telephone and encouraged to send study information and consent to its member list per email or promote the study on their website.

### Data Collection and Measures

The web-based questionnaire was developed by 3 experienced researchers following literature studies and an interdisciplinary discussion with other staff members working at RBUP. The questions were self-developed and subsequently pilot tested independently by 6 health care professionals sampled from our intended study population prior to data collection to ensure face validity. Their feedback did not lead to any changes to the included questions, but the length of the questionnaire was on the verge of being unacceptable. We, therefore, tailored the survey such that participants did not have to complete questions about infant and mental health problems they rarely worked with, as this was considered meaningless and annoying. Consequently, we could only afford to include brief measures and we tailored the survey according to 12 different mental health conditions.

Data were collected between May and September 2018 using the survey platform Confirmit. All infant and child health practitioners working with children younger than 5 years who were able to complete the survey in Norwegian were eligible for the study. Practitioners were provided with written information explaining the purpose of the study and that participation was voluntary. Informed web-based consent was obtained from all participants. After providing informed consent, participants completed the web-based questionnaire, which took, on average, 20 minutes. If no web-based consent was provided, the potential participants could not access the web-based questions. Participants who did not respond to the study invitation after consenting to participate received up to 2 reminders (ie, 1 per week).

First, participants were requested to fill in background information (ie, age, sex, and education). Then, based on the broad categories from the Diagnostic Classification of Mental Health and Developmental Disorders of Infancy and Early Childhood (DC:0-5; [30]), respondents were asked to indicate which of the following 12 different infant and child mental health problems they worked with: (1) parent-child relationship and attachment problems, (2) developmental delays, (3) dysregulation, (4) behavioral problems, (5) social withdrawal and shyness, (6) sleep problems, (7) developmental disorders, (8) breastfeeding and eating problems, (9) anxiety, (10) trauma (developmental and physical), (11) depression, and (12) obsessive behaviors and disorders. Sensory processing disorder, which is a category in DC:0-5, was not included in our list because the disorder is little known and widespread in services and requires specialized services. In addition, we pulled out behavioral difficulties and social withdrawal/shyness: behavioral difficulties because it is subsumed under “Mood disorders” in DC:0-5 and social withdrawal/shyness because it is considered a gateway disorder to other disorders and something many in the Norwegian health care system are trained to look for.

Participants who indicated that they “never” or “rarely” worked with one or several of these mental health problems or that a mental health problem was “not relevant” for their work did not receive any further questions about the perceived usefulness of internet-based parenting interventions for that particular problem. Thereafter, respondents were presented with a brief written introduction, defining and explaining the practical use of self-directed internet-based interventions (see Textbox 1).

**Brief textual information about internet-based interventions adapted and translated from Norwegian.**

**Experience and knowledge of internet-based self-help programs**

- Internet-based interventions are often developed by researchers and clinicians and usually consist of 6-12 weekly consultations. In web-based guidance, parents learn about the challenges they face, do exercises, and receive weekly homework. Interactive content is used and standard internet technology is used.
- The content is based on recognized theories and methods in psychology that are often used by professionals in their work with pregnant women, parents, and children. One of the most common approaches is cognitive behavioral therapy, but psychodynamic and other approaches are also used.
- Internet-based self-help programs do not require any prior knowledge or expertise. You will be trained for 2-4 days and receive guidance from qualified personnel with expertise within the relevant problem area. The guidance takes place over time and as needed.
- Your task as a professional is to support the parents in carrying out the web-based program and solving challenges along the way (eg, low motivation and adaptation of tasks and exercises to the family's own situation). Everything takes place either on the internet or in combination with consultations and is included as part of the ordinary service offering. With web-based guidance or treatment, you will use an average of 10-20 minutes per family per consultation.
- Please answer the following questions based on the information you have now received about internet-based self-help programs.

Perceived usefulness of internet-based parenting interventions was assessed with 1 global item: “*How often do you think internet-based self-help programs can be useful for following infant and child mental health problems in your line of work?*” derived from the global usefulness items in the usefulness scale in the Technology Acceptance Model (ie, “*I would find WriteOne useful in the MBA program*” [20]) and Performance Expectancy scale in the UTAUT (ie, “*I find mobile internet useful in my daily life*” [21]). The answers were scored on a 4-point scale and coded as (0) “*never,*” (1) “*rarely,*” (2) “*sometimes,*” and (3) “*often.*” There was also a “*Not sure/I don’t know*” category. We defined a score ≥2 on any of the mental health conditions as being perceived as “*useful*” by practitioners and leaders herein. The job level was assessed with the question: “*Check the description that best fits your current position.*” The answers were coded as (1) “*top manager (eg, service leader),*” (2) “*middle manager (eg, team leader or project leader),*” (3) “*practitioner (eg, caseworker or therapist),*” and (4) “*other*” (ie, self-employed).

Information on service was assessed from the question: “*Where do you work?*” Responses were coded into 5 health care services: (1) “*daycare centers,*” (2) “*well-baby clinics,*” (3) “*municipal CWS,*” (4) “*CAMHS,*” and (5) “*other services*” (ie, private practice or neonatal intensive care unit).

### Statistical Methods

Descriptive analyses were applied to summarize participant characteristics, missing data, and scores of perceived usefulness for different infant and child mental health problems, including means and standard deviations for continuous variables and frequency counts and percentages for categorical variables. Participants who did not complete the survey, that is, those who had one or more missing data were counted as missing and were compared against those who provided complete data. Participants with missing data were analyzed using chi-square and independent sample two-sided *t* tests for categorical and continuous data, respectively.

Participants in the “*Other*” category were excluded from analyses at job level. The number of participants identifying neither as a top-level, mid-level manager, or practitioner was too low for any comparisons and not of main interest for our research purposes (13/2884, 0.5%). Participants categorized as working in “*other services*” were not included in comparisons of services on perceived usefulness (481/2884, 16.7%). First, other services consisted of a wide range of primary and secondary care services, thereby making any meaningful comparisons practically impossible. Second, we were primarily interested in differences between daycare centers, well-baby clinics, municipal CWS, and CAMHS, as stated in the aims of this study. A one-way between-groups analysis of variance was conducted to explore the impact of leaders and practitioners, and daycare centers, well-baby clinics, municipal CWS, and CAMHS on perceived usefulness of internet-based parenting interventions for different infant and child mental health problems. The data set was assessed for skewness and kurtosis, and a histogram was plotted for outcome variables to check whether they had a normal distribution. All variables were within acceptable range. Posthoc comparisons using Bonferroni correction were used to investigate differences between job level and services. Effect sizes were calculated for systematic differences and expressed as Cohen *d*, which were interpreted as small (0.2), medium (0.5), and large (0.8). The statistical package SPSS version 23 (SPSS Inc) was used for all statistical analyses.

## Results

### Participant Characteristics

A total of 2884 infant and child health leaders and practitioners provided their consent to participate in this study and responded to the web-based questionnaire. Norway has 18 counties as of 2018. We received responses from each county, with the fewest responses from Finnmark (36/2884, 1.3%) and the most responses from Oslo (379/2884, 13.2%). This proportion reflects the population in these counties according to Statistics Norway (2019; ie, 1.4% and 12.7% of the population lived in Finnmark and Oslo during the study period, respectively) [31]. The characteristics of the study population are presented in Table 1. Participants were primarily middle-aged women who had attended college or university for 1-3 years. In addition, most participants reported working in daycare centers, while the least number of participants reported working in CAMHS. Most respondents were practitioners; however, 29.3% (844/2884) were top-level managers.

**Table 1:** Characteristics of the infant and child health leader and practitioner population in this study (N=2884).

Characteristic of the health care professionals		Values	Missing data^a^, values
**Sex, n (%)**			2 (0.1)
	Male	187 (6.5)	N/A^b^
	Female	2695 (93.4)	N/A
Age (years), mean (SD)		46.1 (10.0)	5 (0.2)
**College/University education (years), n (%)**			1 (0.0)
	≤1-3 years	1969 (68.3)	N/A
	≥4-5 years	914 (31.7)	N/A
**Services, n (%)**			57 (2.0)
	Daycare center	1215 (42.1)	N/A
	Well-baby clinic	701 (24.3)	N/A
	Municipal child welfare service	321 (11.1)	N/A
	Child and adolescent mental health clinic	109 (3.8)	N/A
	Other services	481 (16.7)	N/A
**Job level, n (%)**			94 (3.3)
	Top manager (eg, service leader)	844 (29.3)	N/A
	Middle manager (eg, team leader or project leader)	653 (22.6)	N/A
	Practitioner (eg, caseworker or therapist)	1280 (44.4)	N/A
	Other	13 (0.5)	N/A

^a^Participants who did not complete the survey, that is, those who had one or more missing data were counted as missing.

^b^N/A: Not applicable.

### Missing Data

Missing data for participants’ background characteristics are reported in Table 1 above. All respondents were required to fill in demographical data; however, not all respondents received questions about the perceived usefulness of internet-based interventions for all child problems, as explained above. Responses to usefulness were thus conditional in “sometimes” or “often” working with the respective infant or child mental health problems. This also means that each respondent received a varying number of mental health problems for the assessment of the usefulness of internet-based parenting interventions, which partly explains the lesser number of participants used for the analyses in the subsequent tables shown below. Importantly, these were not defined as missing. Only respondents who did not complete all questions were defined as study dropouts. The analysis showed an association between missingness and job level (*χ*_3_^2^=13.27, *P*=.004). Fewer middle managers (169/653, 25.9%) dropped out from the study than practitioners (387/1280, 30.2%) and top managers (271/844, 32.1%). There were no systematic differences in missingness, neither for sex, age, education, or health service (all *P*>.08).

### Overall Perceived Usefulness of Internet-Based Interventions

Participants were asked to indicate how often internet-based parent support interventions could be useful for 12 different infant and child mental health problems. Our results showed that the majority of practitioners and leaders reported that they “*sometimes*” or “*often*” perceived internet-based parenting interventions for the different mental health problems as useful (on a scale from 0 to 3, >76% scored 2=*sometimes* or 3=*often* for 8 out of the 12 conditions, all means>1.61, Table 2). Usefulness of internet-based parenting interventions was rated acceptable for problem areas such as sleep problems, anxiety, and social withdrawal and shyness. More caution was reported toward usefulness for psychiatric problems such as trauma. However, more than half of the participants reported that internet-based interventions for trauma “*sometimes*” or “*often*” could be useful.

**Table 2:** Overall ranking of the perceived usefulness of internet-based programs for infant and child mental health problems.

Rank	Infant and child mental health problem	Participants (n)	Mean (SD)^a^	PU^b^ ≥2, n (%)	Don’t know/not sure (N=2884), n (%)
1	Sleep problems	1115	2.22 (0.67)	992 (89.0)	80 (2.8)
2	Anxiety	1104	2.09 (0.73)	915 (82.9)	92 (3.2)
3	Social withdrawal and shyness	1182	2.07 (0.69)	971 (82.1)	114 (4.0)
4	Dysregulation	1113	2.03 (0.71)	909 (81.7)	79 (2.7)
5	Behavioral problems	1535	2.09 (0.72)	1250 (81.4)	129 (4.5)
6	Breastfeeding and eating problems	938	2.06 (0.73)	761 (81.1)	74 (2.6)
7	Parent-child relationship and attachment problems	1399	2.02 (0.78)	1088 (77.8)	93 (3.2)
8	Developmental delays	1378	1.99 (0.76)	1047 (76.0)	137 (4.7)
9	Depression	847	1.91 (0.74)	621 (73.3)	75 (2.6)
10	Obsessive behaviors and disorders	507	1.89 (0.75)	370 (73.0)	49 (1.7)
11	Developmental disorders	1085	1.91 (0.77)	785 (72.4)	114 (4.0)
12	Trauma	772	1.61 (0.84)	434 (56.2)	85 (2.9)

^a^Scale from 0 to 3 where 0=never, 1=rarely, 2=sometimes, and 3=often.

^b^PU: perceived usefulness.

### Job Level and Usefulness of Internet-Based Parenting Interventions

A one-way between-group analysis of variance was performed to investigate the impact of job level on the perceived usefulness of internet-based parenting interventions for different infant and child mental health problems (Table 3). Participants were divided into 3 groups according to their job level (ie, practitioner, middle manager, and top manager). Overall tests suggested significant differences at the *P*<.05 level for 7 out of 12 mental health problems: (1) behavioral problems, (2) parent-child relationship and attachment problems, (3) social withdrawal and shyness, (4) trauma, (5) dysregulation, (6) developmental delays, and (7) developmental disorders. Despite reaching statistical significance, actual mean differences between the groups were small. Posthoc comparisons using the Bonferroni adjustment indicated an overall tendency that most differences were between practitioners and top-level managers (Multimedia Appendix 1). Top-level managers found internet-based parenting interventions more useful than practitioners in 6 areas: (1) behavioral problems, (2) parent-child relationship and attachment problems, (3) social withdrawal and shyness, (4) trauma, (5) developmental delays, and (6) developmental disorders. In addition, posthoc comparisons indicated that there were differences in the mean scores between practitioners and middle managers for developmental delays in that middle managers expressed more positive attitudes to internet-based interventions than practitioners. All effect sizes, calculated using Cohen *d*, were small (all *d*<0.32, all *P*<.02).

**Table 3:** Comparisons of the perceived usefulness of internet-based interventions for 12 different infant and child mental health problems by practitioners at different job levels (employment status) by using one-way analysis of variance.

Mental health problems, job level of health care practitioner		Participants (n)	Mean (SD)	*F (df)*	*P* value
**Breastfeeding and eating problems**				0.44 (2)	.64
	Practitioner	490	2.04 (0.72)		
	Middle manager	231	2.09 (0.71)		
	Top manager	215	2.08 (0.76)		
**Anxiety**				0.61 (2)	.55
	Practitioner	526	2.07 (0.73)		
	Middle manager	256	2.11 (0.75)		
	Top manager	319	2.12 (0.70)		
**Behavioral problems**				5.34 (2)	.005^a^
	Practitioner	675	2.03 (0.75)		
	Middle manager	386	2.09 (0.73)		
	Top manager	472	2.17 (0.66)		
**Depression**				2.88 (2)	.06
	Practitioner	402	1.86 (0.77)		
	Middle manager	203	1.92 (0.71)		
	Top manager	242	2.00 (0.73)		
**Parent-child relationship and attachment problems**				8.01 (2)	<.001^b^
	Practitioner	679	1.94 (0.79)		
	Middle manager	329	2.02 (0.79)		
	Top manager	388	2.14 (0.74)		
**Social withdrawal and shyness**				4.60 (2)	<.001^b^
	Practitioner	526	2.00 (0.69)		
	Middle manager	304	2.10 (0.72)		
	Top manager	350	2.14 (0.64)		
**Sleep problems**				0.36 (2)	.70
	Practitioner	550	2.22 (0.67)		
	Middle manager	268	2.25 (0.68)		
	Top manager	294	2.20 (0.66)		
**Trauma**				4.18 (2)	.02^c^
	Practitioner	434	1.54 (0.83)		
	Middle manager	164	1.65 (0.88)		
	Top manager	174	1.75 (0.82)		
**Obsessive behaviors and disorders**				1.26 (2)	.29
	Practitioner	287	1.84 (0.75)		
	Middle manager	100	1.95 (0.67)		
	Top manager	120	1.94 (0.78)		
**Dysregulation**				3.43 (2)	.03^c^
	Practitioner	562	1.97 (0.73)		
	Middle manager	252	2.08 (0.69)		
	Top manager	296	2.08 (0.67)		
**Developmental delays**				14.79 (2)	<.001^b^
	Practitioner	597	1.87 (0.77)		
	Middle manager	355	2.06 (0.76)		
	Top manager	425	2.11 (0.74)		
**Developmental disorders**				6.56 (2)	<.001^b^
	Practitioner	495	1.81 (0.78)		
	Middle manager	263	1.97 (0.78)		
	Top manager	324	2.00 (0.74)		

^a^This value was significant at *P*<.01.

^b^This value was significant at *P*<.001.

^c^This value was significant at *P*<.05.

### Services and Usefulness of Internet-Based Parenting Interventions

A one-way between-group analysis of variance was performed to investigate the impact of services on the perceived usefulness of internet-based parenting interventions for different infant and child mental health problems. Overall tests between health care services suggested systematic differences at the *P*<.05 level for all mental health problems, except breastfeeding and eating problems (Table 4). However, as with the job level, actual differences in the mean scores between the services were small. Posthoc tests with Bonferroni corrections showed a few small significant differences between health services (Multimedia Appendix 2). An overall tendency was that most differences were between daycare centers and the 3 remaining services. Daycare centers considered internet-based parenting interventions as more useful for behavioral problems, parent-child relationship and attachment problems, social withdrawal and shyness, trauma, dysregulation, developmental delays, and developmental disorders than the other services. All effect sizes, calculated using Cohen *d*, were small to moderate (all *d*<0.69, all *P*<.006).

**Table 4:** Comparisons of the perceived usefulness of internet-based interventions by different services for 12 different infant and child mental health problems by using one-way analysis of variance.

Mental health problems, service		Participants (n)	Mean (SD)	*F (df)*	*P* value
**Breastfeeding and eating problems**				1.46 (3)	.23
	CAMHS^a^	53	2.15 (0.57)		
	Daycare centers	305	2.12 (0.73)		
	Well-baby clinics	369	2.02 (0.71)		
	Municipal CWS^b^	55	2.11 (0.69)		
**Anxiety**				3.08 (3)	.03^c^
	CAMHS	54	2.24 (0.55)		
	Daycare centers	465	2.15 (0.73)		
	Well-baby clinics	250	2.12 (0.72)		
	Municipal CWS	148	1.97 (0.71)		
**Behavioral problems**				5.33 (3)	.001^d^
	CAMHS	59	2.03 (0.59)		
	Daycare centers	720	2.18 (0.72)		
	Well-baby clinics	344	2.09 (0.69)		
	Municipal CWS	193	1.96 (0.76)		
**Depression**				3.95 (3)	.008^d^
	CAMHS	53	1.92 (0.58)		
	Daycare centers	330	2.04 (0.75)		
	Well-baby clinics	155	1.86 (0.75)		
	Municipal CWS	147	1.82 (0.73)		
**Parent-child relationship and attachment problems**				12.91 (3)	<.001^e^
	CAMHS	65	1.68 (0.69)		
	Daycare centers	541	2.18 (0.76)		
	Well-baby clinics	349	2.00 (0.77)		
	Municipal CWS	197	1.92 (0.77)		
**Social withdrawal and shyness**				7.06 (3)	<.001^e^
	CAMHS	53	2.09 (0.53)		
	Daycare centers	561	2.16 (0.68)		
	Well-baby clinics	271	2.06 (0.68)		
	Municipal CWS	125	1.86 (0.66)		
**Sleep problems**				4.07 (3)	.007^d^
	CAMHS	56	2.27 (0.59)		
	Daycare centers	426	2.21 (0.68)		
	Well-baby clinics	369	2.31 (0.63)		
	Municipal CWS	105	2.08 (0.66)		
**Trauma**				5.51 (3)	.001^d^
	CAMHS	62	1.60 (0.78)		
	Daycare centers	194	1.81 (0.87)		
	Well-baby clinics	150	1.47 (0.86)		
	Municipal CWS	190	1.55 (0.76)		
**Obsessive behaviors and disorders**				2.65 (3)	.049^c^
	CAMHS	45	2.04 (0.64)		
	Daycare centers	149	1.96 (0.77)		
	Well-baby clinics	97	1.77 (0.68)		
	Municipal CWS	102	1.78 (0.74)		
**Dysregulation**				8.15 (3)	<.001^e^
	CAMHS	52	1.88 (0.76)		
	Daycare centers	418	2.11 (0.68)		
	Well-baby clinics	321	2.11 (0.64)		
	Municipal CWS	133	1.82 (0.69)		
**Developmental delays**				17.95 (3)	<.001^e^
	CAMHS	57	1.88 (0.68)		
	Daycare centers	652	2.16 (0.76)		
	Well-baby clinics	316	1.84 (0.74)		
	Municipal CWS	167	1.84 (0.67)		
**Developmental disorders**				10.79 (3)	<.001^e^
	CAMHS	61	2.07 (0.70)		
	Daycare centers	466	2.04 (0.78)		
	Well-baby clinics	228	1.76 (0.74)		
	Municipal CWS	157	1.75 (0.70)		

^a^CAMHS: child and adolescent mental health clinics.

^b^CWS: child welfare services.

^c^This value was significant at *P*<.05.

^d^This value was significant at *P*<.01.

^e^This value was significant at *P*<.001.

## Discussion

### Overview of the Findings

Despite the rapid development of e-mental health services and the promising evidence for their utility [32], less attention has been paid to whether practitioners are positive toward internet-based interventions and would find these useful in their practice. Therefore, in this cross-sectional study of 2884 infant and child health leaders and practitioners, we aimed to investigate health professionals’ perceived usefulness of e-mental health programs and identify the key areas in which they consider new e-mental health services to be useful, as well as differences between service practitioners and leaders, and different prenatal, infant, and child health care services for children aged 0-5 years. The results showed that a majority reported that they would sometimes or often find internet-based parenting interventions for different infant and child mental health problems as useful. Usefulness of internet-based interventions was rated acceptable for sleep problems, anxiety, and social withdrawal and shyness, whereas fewer reported that it would be useful for psychiatric problems such as obsessive disorders or trauma (eg, child maltreatment). Moreover, there were a few but small differences in the perceived usefulness between service leaders and practitioners (all effect sizes<0.32, all *P*<.02) and small-to-moderate differences between daycare centers, well-baby clinics, CWS, and CAMHS (all effect sizes<0.69, all *P*<.006).

Our findings are in line with results of previous studies that show that practitioners generally consider internet-based interventions useful but that attitudes may range from skepticism to positivity for prevention and treatment of mild-to-moderate problems [24,25,33]. These studies have also found that health personnel hold more negative views toward their usefulness for severe disorders. In our study, we compared a broad range of different infant and child mental health problems, which, to our knowledge, has not been done before. Participants had a positive attitude toward the use of internet-based interventions for infant and child health problems such as sleep problems, anxiety, social withdrawal, and dysregulation. The results revealed a more ambivalent attitude towards the use of e-mental health services for problem areas such as developmental delays and trauma. However, even for such problem areas, most practitioners recognized the potential of e-mental health interventions and seemed to be aware of its usefulness. With respect to the more ambivalent attitude of eHealth for some infant and child health problems, a possible explanation for this is that practitioners may perceive these problems as more severe and in need of different kinds of treatments. For example, Stallard et al [24] have shown that concerns about using eHealth with children and adolescents has 4 themes: limited potential, risk management, support and understanding, and lack of therapeutic relationship. Issues relating to the importance of therapeutic alliance are also found in adult literature [34]. Such concerns may apply more to disorders that are considered as clinically more severe and where there is a need for more interdisciplinary and frequent follow-up than mild-to-moderate problems. Furthermore, lack of knowledge about eHealth among practitioners may also be a possible explanation for the more ambivalent attitude to using e-mental health services for some problems.

To our knowledge, this study is the first to compare differences in the perceived usefulness of internet-based interventions for different infant and child mental health problems between leaders and practitioners and between different infant and child health services. Our results showed that top-level managers found internet-based interventions more useful than practitioners for several infant and child mental health problems. As this study is the first to compare differences in perceived usefulness between leaders and practitioners, the causes of the difference in opinion between the 2 occupational levels are not clearly identified. However, the UTAUT model hypothesizes that individual-level contextual factors such as gender, age, actual experience, and voluntariness of use would moderate the effect of behavioral intention [21]. Hence, potential differences in these variables among leaders and practitioners (ie, leaders are often older, more experienced, and exhibit more voluntariness of use) may contribute to explain why top-level managers found internet-based interventions more useful than practitioners on several infant and child mental health problems.

Previous research has shown that implementation leadership is a critical factor for organizational changes [35]. Hence, leadership may either promote or inhibit the adoption of e-mental health in services [36]. Even if practitioners are open to e-mental health, effective leadership may help to support implementation climate and efforts [35]. Some leaders may develop plans, anticipate and address implementation challenges, and have clear priorities and expectations (ie, proactive leadership), while others may give up when they face obstacles or fail to address challenges effectively (ie, nonperseverant leadership, [35]). A transformational leadership (ie, proactive) style seems to be important for the adoption of information technology [27], and previous studies have reported similar experiences with implementation of an internet-based postpartum depression intervention that show the importance of leadership [26].

According to the UTAUT model, higher-level contextual factors such as organization attributes would also influence technology acceptance and use [21]. Differences in perceived usefulness of internet-based interventions could therefore be expected for people working in different fields of the health care system. Contrary to this, we found few differences in perceived usefulness between infant and child health services. Although there are many similarities between infant and child health services [37], there may still be differences in how acceptable, feasible, and suitable e-mental health services can be. Hence, our findings make it important to further examine what opportunities exist within services, as there are also challenges and opportunities unique to each [35]. Some services may perceive that e-mental health services for a specific condition can be useful, but organizational conditions can still make them unworkable or inappropriate. This can, for example, in line with the UTAUT model be more structural and organizational conditions such as climate, organizational culture, leadership or grant schemes for parental support interventions, and other (policy) guidelines from official health authorities. As a future direction, such things may be important to consider from the outset before developing e-mental health services to increase the likelihood of new interventions being taken up and used in services.

Overall, infant and child health practitioners’ attitudes toward internet-based interventions were positive, suggesting that many practitioners may be open to taking advantage of internet-based interventions. Only a minor proportion of those who could benefit from evidence-based parenting programs seem to receive these. Hence, our findings are encouraging, considering the potential of technology to expand the delivery portfolio to overcome barriers associated with face-to-face delivery and increase the availability and accessibility of e-mental health interventions to bridge the gaps in the provision of care. However, despite the general positivity toward such interventions, few web-based interventions are available for families with children aged 0-5 years. Programs for this age group are also mostly for disruptive child behaviors [15], and there appears to be only few programs that have been studied and made available in non-English languages. Hence, additional efforts are needed to develop, implement, and disseminate interventions for families with infants and young children.

### Strengths and Limitations

This study has both strengths and limitations that should be recognized. To our knowledge, this study was the first to address health professionals’ perceived usefulness of e-mental health programs for different infant and child problems and to compare different services and job levels. The advantage is that this study provides new insights. However, the disadvantage is that there are no comparable studies to rely on. For that reason, we may have omitted relevant aspects. Another notable strength is the large number of participants as well as a national sample consisting of practitioners from all major infant and child health services in Norway, likely to be targeted in dissemination efforts. However, as the study was web-based and practitioners were only contacted via email or social media, a selection bias may have been introduced. Practitioners facing greater practical barriers to the use of computers and internet (ie, lower computer fluency and reduced access to technology at work) may have been more reluctant to participate in the study. This has also been shown to have a negative impact on people’s perceived usefulness of information technology [38]. However, 1 study suggested that prevalence estimates of exposure and outcome but not estimates of exposure-outcome associations are biased due to self-selection. Hence, it is important to bear in mind that selection bias does not necessarily influence results much when associations between variables are investigated [39]. Other drawbacks associated with web-based surveys are also important to keep in mind such as researchers cannot check whether participants have understood and interpreted the questions in the same way as the researchers meant. Further, participants cannot elaborate on their answers; therefore, we may have potentially missed nuances that would have yielded valuable insights. However, the web-based questionnaire was pilot tested independently by 6 health care professionals sampled from our intended study population, prior to data collection so as to minimize the risk for misinterpretation. Another limitation was that perceived usefulness of internet-based interventions was only assessed with 1 global item. However, to our knowledge, no general instruments exist for measuring perceived usefulness across different types of information technology, as these always must be modified to accommodate the specifics of the attitude object that is being evaluated. Thus, conducting studies with more extensive questions may have allowed for further insight into the topic, although our overall results are also consistent with those of previous studies. This supports our findings and indicates that a global single-item assessment of perceived usefulness is fully possible. However, using more questions may have added more variation, thereby making the results between different job levels and health and social services more distinguishable. This was not practically possible for our study, without making the survey potentially unacceptable and too time-consuming to complete. Other limitations include the cross-sectional nature of this study as well as multiple testing. Even when adjusting for multiple testing, it is possible that the significant results of some items may be due to chance. Thus, any significant finding must be interpreted with caution. Furthermore, the study was conducted in Norway. Thus, the results may not be applicable to countries with widely different health care systems, which may potentially limit the generalizability of our study findings. A final limitation may be that even the provision of brief textual information about e-mental health services can influence people’s attitudes toward e-mental health [40,41], which may have affected participants’ evaluations in our study.

### Future Research

Future research should include more detailed questions about factors that could influence perceived usefulness, for example, general openness to new treatments, organizational support, and practical problems and barriers that limit successful implementation. For e-mental health to have the large public health impact that it is often praised for, there is a need for improving the translation of e-mental health research into clinical practice. Therefore, there is also a need for more research on cocreation of interventions adapted to both services and parents’ needs, as well as conditions in clinical practice such as lack of time, resources, and low visibility [17]. At last, considering the COVID-19 pandemic and the consequent increase in the use of digital solutions, it can also be useful to conduct a new study as this crisis may have affected health professionals’ perceived usefulness of e-mental health programs for different infant and child problems.

### Conclusion

This study shows that internet-based interventions for different infant and child mental health problems within services such as daycare centers, well-baby clinics, municipal CWS, and CAMHS are sometimes or often perceived as useful. These are encouraging findings and support the continued exploration of internet-based mental health interventions as way to improve parental support. In turn, these insights may inform processes of technological development, clinical use, and organizational implementation of internet-based interventions.

## Appendix

Multimedia Appendix 1 Results from multiple comparisons utilizing the posthoc Bonferroni test_job level.

Multimedia Appendix 2 Results from multiple comparisons utilizing the posthoc Bonferroni test_service.

## Abbreviations

CAMHS: child and adolescent mental health clinics
CWS: child welfare services
DC 0-5: Diagnostic Classification of Mental Health and Developmental Disorders of Infancy and Early Childhood
RBUP: Regional Centre for Child and Adolescent Mental Health
UTAUT: Unified Theory of Acceptance and Use of Technology
