# Supplementary material for: Health Personnel’s Perceived Usefulness of Internet-Based Interventions for Parents of Children Younger Than 5 Years: Cross-Sectional Web-Based Survey Study
Source: JMIR Ment Health. 2020 Nov 18;7(11):e15149. doi: 10.2196/15149 (PMC7710450; doi:10.2196/15149)
Supplement: Multimedia Appendix 1 [file mental_v7i11e15149_app1.docx]

| Multimedia Appendix 1. Results from multiple comparisons utilizing the post-hoc Bonferroni test. | | | | | | |
| --- | --- | --- | --- | --- | --- | --- |
| *Dependent variable* | *Job level* | *Comparator* | *Mean difference* | *SE* | *P* | *d* |
| Breastfeeding and/or eating problems | Employee | Middle manager | -0.05 | 0.06 | 1.000 |  |
|  |  | Top manager | -0.04 | 0.06 | 1.000 |  |
|  | Middle manger | Top manager | 0.01 | 0.07 | 1.000 |  |
| Anxiety | Employee | Middle manager | -0.04 | 0.06 | 1.000 |  |
|  |  | Top manager | -0.05 | 0.05 | .976 |  |
|  | Middle manger | Top manager | -0.01 | 0.06 | 1.000 |  |
| Behavioural problem | Employee | Middle manager | -0.06 | 0.05 | .541 |  |
|  |  | Top manager | -0.14 | 0.04 | .003* | .198 |
|  | Middle manger | Top manager | -0.08 | 0.05 | .323 |  |
| Depression | Employee | Middle manager | -0.07 | 0.06 | .917 |  |
|  |  | Top manager | -0.14 | 0.06 | .051 |  |
|  | Middle manger | Top manager | -0.08 | 0.07 | .794 |  |
| Parent-child relationship and/or attachment problems | Employee | Middle manager | -0.08 | 0.05 | .417 |  |
|  |  | Top manager | -0.20 | 0.05 | <.001* | .261 |
|  | Middle manger | Top manager | -0.12 | 0.06 | .116 |  |
| Social withdrawal and/or shyness | Employee | Middle manager | -0.1 | 0.05 | .142 |  |
|  |  | Top manager | -0.14 | 0.05 | .012* | .209 |
|  | Middle manger | Top manager | -0.04 | 0.05 | 1.000 |  |
| Sleep problems | Employee | Middle manager | -0.03 | 0.05 | 1.000 |  |
|  |  | Top manager | 0.01 | 0.05 | 1.000 |  |
|  | Middle manger | Top manager | 0.05 | 0.06 | 1.000 |  |
| Trauma | Employee | Middle manager | -0.12 | 0.08 | .401 |  |
|  |  | Top manager | -0.21 | 0.08 | .016* | .254 |
|  | Middle manger | Top manager | -0.09 | 0.09 | .901 |  |
| Obsessive behaviors and/or disorders | Employee | Middle manager | -0.11 | 0.09 | .610 |  |
|  |  | Top manager | -0.10 | 0.08 | .627 |  |
|  | Middle manger | Top manager | 0.01 | 0.10 | 1.000 |  |
| Dysregulation | Employee | Middle manager | -0.11 | 0.05 | .131 |  |
|  |  | Top manager | -0.11 | 0.05 | .077 |  |
|  | Middle manger | Top manager | -0.01 | 0.06 | 1.000 |  |
| Developmental delays | Employee | Middle manager | -0.19 | 0.05 | <.001* | .249 |
|  |  | Top manager | -0.24 | 0.05 | <.001* | .318 |
|  | Middle manger | Top manager | -0.05 | 0.05 | 1.000 |  |
| Developmental disorders | Employee | Middle manager | -0.15 | 0.06 | .030 |  |
|  |  | Top manager | -0.18 | 0.06 | .003* | .249 |
|  | Middle manger | Top manager | -0.03 | 0.06 | 1.000 |  |
| * Mean difference significant at the 0.0166 level. | | | | | | |
