# Supplementary material for: Health Personnel’s Perceived Usefulness of Internet-Based Interventions for Parents of Children Younger Than 5 Years: Cross-Sectional Web-Based Survey Study
Source: JMIR Ment Health. 2020 Nov 18;7(11):e15149. doi: 10.2196/15149 (PMC7710450; doi:10.2196/15149)
Supplement: Multimedia Appendix 2 [file mental_v7i11e15149_app2.docx]

| Multimedia Appendix 2. Results from multiple comparisons utilizing the post-hoc Bonferroni test. | | | | | | |
| --- | --- | --- | --- | --- | --- | --- |
| *Dependent variable* | *Service* | *Comparator* | *Mean difference* | *SE* | *P* | *d* |
| Breastfeeding and/or eating problems | CAMHS | Daycare centres | 0.03 | 0.11 | 1.000 |  |
|  |  | Well-baby clinics | 0.13 | 0.10 | 1.000 |  |
|  |  | Municipal CWS | 0.04 | 0.14 | 1.000 |  |
|  | Daycare centres | Well-baby clinics | 0.10 | 0.05 | .385 |  |
|  |  | Municipal CWS | 0.01 | 0.10 | 1.000 |  |
|  | Well-baby clinics | Municipal CWS | -0.09 | 0.10 | 1.000 |  |
| Anxiety | CAMHS | Daycare centres | 0.09 | 0.10 | 1.000 |  |
|  |  | Well-baby clinics | 0.12 | 0.11 | 1.000 |  |
|  |  | Municipal CWS | 0.27 | 0.11 | .095 |  |
|  | Daycare centres | Well-baby clinics | 0.03 | 0.06 | 1.000 |  |
|  |  | Municipal CWS | 0.18 | 0.07 | .038 |  |
|  | Well-baby clinics | Municipal CWS | 0.15 | 0.07 | .261 |  |
| Behavioural problems | CAMHS | Daycare centres | -0.14 | 0.10 | .835 |  |
|  |  | Well-baby clinics | -0.05 | 0.10 | 1.000 |  |
|  |  | Municipal CWS | 0.08 | 0.11 | 1.000 |  |
|  | Daycare centres | Well-baby clinics | 0.09 | 0.05 | .336 |  |
|  |  | Municipal CWS | 0.22 | 0.06 | .001* | .298 |
|  | Well-baby clinics | Municipal CWS | 0.13 | 0.06 | .267 |  |
| Depression | CAMHS | Daycare centres | -0.12 | 0.11 | 1.000 |  |
|  |  | Well-baby clinics | 0.06 | 0.12 | 1.000 |  |
|  |  | Municipal CWS | 0.10 | 0.12 | 1.000 |  |
|  | Daycare centres | Well-baby clinics | 0.18 | 0.07 | .077 |  |
|  |  | Municipal CWS | 0.22 | 0.07 | .016 |  |
|  | Well-baby clinics | Municipal CWS | 0.04 | 0.08 | 1.000 |  |
| Parent-child relationship and/or attachment problems | CAMHS | Daycare centres | -0.50 | 0.10 | <.001* | .693 |
|  |  | Well-baby clinics | -0.32 | 0.10 | .010 |  |
|  |  | Municipal CWS | -0.24 | 0.11 | .157 |  |
|  | Daycare centres | Well-baby clinics | 0.18 | 0.05 | .004* | .236 |
|  |  | Municipal CWS | 0.26 | 0.06 | <.001* | .341 |
|  | Well-baby clinics | Municipal CWS | 0.08 | 0.07 | 1.000 |  |
| Social withdrawal and/or shyness | CAMHS | Daycare centres | -0.07 | 0.10 | 1.000 |  |
|  |  | Well-baby clinics | 0.04 | 0.10 | 1.000 |  |
|  |  | Municipal CWS | 0.23 | 0.11 | .216 |  |
|  | Daycare centres | Well-baby clinics | 0.11 | 0.05 | .205 |  |
|  |  | Municipal CWS | 0.30 | 0.07 | <.001* | .448 |
|  | Well-baby clinics | Municipal CWS | 0.19 | 0.07 | .050 |  |
| Sleep problems | CAMHS | Daycare centres | 0.05 | 0.09 | 1.000 |  |
|  |  | Well-baby clinics | -0.05 | 0.09 | 1.000 |  |
|  |  | Municipal CWS | 0.19 | 0.11 | .457 |  |
|  | Daycare centres | Well-baby clinics | -0.10 | 0.05 | .181 |  |
|  |  | Municipal CWS | 0.14 | 0.07 | .321 |  |
|  | Well-baby clinics | Municipal CWS | 0.24 | 0.07 | .006* | .356 |
| Trauma | CAMHS | Daycare centres | -0.21 | 0.12 | .464 |  |
|  |  | Well-baby clinics | 0.12 | 0.12 | 1.000 |  |
|  |  | Municipal CWS | 0.05 | 0.12 | 1.000 |  |
|  | Daycare centres | Well-baby clinics | 0.34 | 0.09 | .001* | .394 |
|  |  | Municipal CWS | 0.26 | 0.08 | .012 |  |
|  | Well-baby clinics | Municipal CWS | -0.07 | 0.09 | 1.000 |  |
| Obsessive behaviors and/or disorders | CAMHS | Daycare centres | 0.08 | 0.12 | 1.000 |  |
|  |  | Well-baby clinics | 0.27 | 0.13 | .236 |  |
|  |  | Municipal CWS | 0.26 | 0.13 | .279 |  |
|  | Daycare centres | Well-baby clinics | 0.19 | 0.09 | .301 |  |
|  |  | Municipal CWS | 0.18 | 0.09 | .368 |  |
|  | Well-baby clinics | Municipal CWS | -0.01 | 0.10 | 1.000 |  |
| Dysregulation | CAMHS | Daycare centres | -0.23 | 0.10 | .138 |  |
|  |  | Well-baby clinics | -0.22 | 0.10 | .156 |  |
|  |  | Municipal CWS | 0.07 | 0.11 | 1.000 |  |
|  | Daycare centres | Well-baby clinics | 0.00 | 0.05 | 1.000 |  |
|  |  | Municipal CWS | 0.29 | 0.07 | <.001* | .422 |
|  | Well-baby clinics | Municipal CWS | 0.29 | 0.07 | <.001* | .434 |
| Developmental delays | CAMHS | Daycare centres | -0.28 | 0.10 | 0.037 |  |
|  |  | Well-baby clinics | 0.04 | 0.11 | 1.000 |  |
|  |  | Municipal CWS | 0.03 | 0.11 | 1.000 |  |
|  | Daycare centres | Well-baby clinics | 0.32 | 0.05 | <.001* | .428 |
|  |  | Municipal CWS | 0.31 | 0.06 | <.001* | .449 |
|  | Well-baby clinics | Municipal CWS | -0.01 | 0.07 | 1.000 |  |
| Developmental disorders | CAMHS | Daycare centres | 0.03 | 0.10 | 1.000 |  |
|  |  | Well-baby clinics | 0.30 | 0.11 | .032 |  |
|  |  | Municipal CWS | 0.32 | 0.11 | .029 |  |
|  | Daycare centres | Well-baby clinics | 0.28 | 0.06 | <.001* | .369 |
|  |  | Municipal CWS | 0.29 | 0.07 | <.001* | .392 |
|  | Well-baby clinics | Municipal CWS | 0.02 | 0.08 | 1.000 |  |
| * Mean difference is significant at the 0.0083 level. | | | | | | |
